# Spatial signature of low-frequency network changes accounts for pallidal stimulation outcome in cervical dystonia

**DOI:** 10.1016/j.ebiom.2026.106140

**Published:** 2026-01-28

**Authors:** Bahne H. Bahners, Roxanne Lofredi, Hannah Voss, Ana Luísa de Almeida Marcelino, Lukas L. Goede, Lucia K. Feldmann, Alfons Schnitzler, Tilmann H. Sander, Esther Florin, Andrea A. Kühn

**Affiliations:** a Institute of Clinical Neuroscience and Medical Psychology, Medical Faculty and University Hospital Düsseldorf, Heinrich Heine University Düsseldorf, Germany; b Center for Movement Disorders and Neuromodulation, Department of Neurology, Medical Faculty and University Hospital Düsseldorf, Heinrich Heine University Düsseldorf, Germany; c Center for Brain Circuit Therapeutics, Department of Neurology, Brigham & Women's Hospital, Harvard Medical School, Boston, MA, USA; d Movement Disorders and Neuromodulation Unit, Department of Neurology, Charité – Universitätsmedizin Berlin, Corporate Member of Freie Universität Berlin and Humboldt- Universität zu Berlin, Germany; e Berlin Institute of Health, Anna-Louisa-Karsch-Straße 2, 10178, Berlin, Germany; f Physikalisch-Technische Bundesanstalt, Abbestraße 2-12, 10587, Berlin, Germany; g NeuroCure, Charité – Universitätsmedizin Berlin, Berlin, Germany; h Deutsches Zentrum für Neurodegenerative Erkrankungen (DZNE), Berlin, Germany

**Keywords:** Magnetoencephalography, Globus pallidus internus, Electrophysiology effect mapping, Network mapping, Biomarker

## Abstract

**Background:**

Pallidal deep brain stimulation (DBS) has remarkable effects in patients with cervical dystonia. Yet, its neurophysiological mechanisms are not fully resolved to date. Converging evidence suggests that pallidal DBS modulates sensorimotor and cerebellar network activity in dystonia, possibly by disrupting pathologically enhanced low-frequency oscillations in the basal ganglia. Still, anatomical and electrophysiological findings have rarely been linked, and it is unclear whether oscillatory changes occur in the same network identified in neuroimaging studies.

**Methods:**

In this cross-sectional study, we investigate the effects of pallidal DBS in patients with cervical dystonia using magnetoencephalography recordings on and off stimulation. We correlated DBS outcomes to the whole-cortex pattern of DBS-induced power changes in each cortical vertex.

**Findings:**

This analysis revealed a distinct low-frequency electrophysiological signature that accounted for significant amounts of variance in DBS improvements across the cohort. The signature was characterised by negative peaks within the supplementary motor area and the motor cortex as well as positive peaks in prefrontal and cerebellar areas.

**Interpretation:**

Our study sheds light on the cortical and cerebellar effects of pallidal DBS on a whole-cortex level and puts emphasis on low-frequency power modulation as a mechanism of effective stimulation beyond the basal ganglia in patients with cervical dystonia. Our findings might inform DBS programming and targeting as well as non-invasive stimulation strategies in the future.

**Funding:**

10.13039/501100001659Deutsche Forschungsgemeinschaft (DFG, German Research Foundation)—Project-ID 424778381—TRR 295.

**Research in context**

**Evidence before this study**

Previous neurophysiological and imaging studies have suggested that pallidal deep brain stimulation (DBS) suppresses pathologically enhanced low-frequency oscillations and modulates sensorimotor and cerebellar networks in dystonia. However, electrophysiological and anatomical findings have rarely been linked, and it remained unclear whether oscillatory changes occur within the same networks identified in neuroimaging studies.

**Added value of this study**

By acquiring magnetoencephalography recordings in patients with cervical dystonia during DBS on and off conditions, we identified a distinct cortical and cerebellar low-frequency signature that was closely associated with clinical outcomes. Our study integrates anatomical and electrophysiological domains and provides novel evidence for a whole-cortex response pattern that explains significant variance in stimulation outcomes.

**Implications of all the available evidence**

These findings emphasise dystonia as a network disorder and highlight low-frequency modulation across motor and cerebellar regions as a key mechanism of pallidal DBS. Low-frequency suppression in the supplementary motor and motor cortices, along with synchronisation in frontal and cerebellar areas, may serve as clinically relevant biomarkers and inform DBS programming, targeting strategies, and non-invasive stimulation approaches.

## Introduction

Pallidal deep brain stimulation (DBS) is an effective treatment option for patients with dystonia.^1^^,^^2^ However, the clinical outcome of pallidal DBS in dystonia is variable,^3^ and the underlying mechanisms are not fully understood. One proposed electrophysiological mechanism, supported by various studies, is the suppression of low-frequency neuronal activity that is pathologically enhanced^4^, ^5^, ^6^ within multiple nodes in the basal ganglia thalamo-cortical loop.^7^ Indeed, it is widely acknowledged, that dystonia is a network disorder^7^, ^8^, ^9^ and may respond to the stimulation at different network levels beyond the basal ganglia including cerebellar nodes.^7^^,^^10^, ^11^, ^12^, ^13^, ^14^, ^15^, ^16^

To capture the DBS-induced neuronal network changes in the *temporal* and *spatial* domain, different methods have been employed. In the *neurophysiological* domain, pathologically enhanced low-frequency activity (<12 Hz) has been identified as a key local and network-level marker that should be targeted for symptom improvement in dystonia.^5^^,^^6^^,^^12^^,^^17^, ^18^, ^19^, ^20^, ^21^, ^22^ Meanwhile, pallidal beta oscillations have been attributed to DBS-induced side effects such as slowness of movements in patients with dystonia^23^ and bradykinesia in Parkinson's disease.^19^^,^^24^ Besides DBS-induced suppression of low-frequency activity observed in pallidal local field potential (LFP) recordings,^5^ scalp EEG recordings have revealed an effect of pallidal DBS on cortical low-frequency activity.^6^ In simultaneous pallidal LFP and EEG recordings pallido-cortical low-frequency coherence was reduced, while coherence in the beta band seemed unaffected by pallidal DBS in dystonia.^5^

On the *neuroimaging* side, DBS-induced changes in dystonia localise to frontal regions including the supplementary motor area (SMA), the sensorimotor cortex but also the cerebellum^25^, ^26^, ^27^, ^28^, ^29^, ^30^, ^31^ and correlate with the magnitude of the clinical DBS response.^31^ The DBS response network identified using fMRI network mapping, includes positive connectivity from the stimulation site to cerebellum and midbrain, while somato-motor areas were negatively connected.^11^ Overall, the restoration of these local and long-range fMRI connectivity patterns toward the healthy network state appears to be relevant for achieving optimal DBS outcomes.^9^

Thus, in an attempt to interpret imaging and neurophysiological findings together, one could postulate that pallidal DBS modulates sensorimotor and cerebellar network activity in dystonia, possibly by disrupting pathologically enhanced low-frequency oscillations. However, none of the above studies has linked these anatomical and electrophysiological findings. One method to study cortical DBS effects in both the electrophysiological and anatomical domain is magnetoencephalography (MEG).^32^, ^33^, ^34^ MEG is particularly well-suited to spatially resolve the cortical sources of neuronal activity at high temporal resolution.

Here we investigate the whole-cortex effects and side effects of pallidal DBS in 16 patients with cervical dystonia using MEG recordings on and off stimulation. To interpret findings together along *anatomical* and *electrophysiological* domains, we apply an established method extending on previous work in fMRI, MEG and EEG.^34^^,^^35^ We correlate individual DBS outcomes to the oscillatory *cortical pattern* of low-frequency power *changes* in each cortical vertex. The resulting ‘optimal’ map of correlation coefficients represents the cortical pattern of changes that beneficial stimulation settings would elicit. This ‘optimal’ map allowed us to estimate significant amounts of variance in DBS improvements and was characterised by low frequency power suppression within the SMA and the motor cortex as well as low-frequency synchronisation in prefrontal and cerebellar areas.

## Methods

### Patients

We recruited 16 patients (11 female, mean age: 64 ± 7 years) who had received bilateral globus pallidus internus (GPi) DBS for treatment-refractory cervical dystonia (mean treatment duration: 7 years and 7 months, range: 9 months to 16 years) at two German DBS centres (Berlin, n = 13; Düsseldorf, n = 3). Besides cervical dystonia, an adjacent body part was affected in four patients, granting the diagnosis of segmental dystonia (see Table 1). These symptoms occurred in the left upper limb in three patients and the right shoulder girdle in one patient. Further clinical details are summarised in Table 1. Only a subset of patients in the present cohort underwent genetic testing, and none carried pathogenic variants in known dystonia genes. Future studies with larger cohorts may systematically investigate whether specific genetic forms of dystonia exhibit distinct oscillatory network signatures, as recent work has reported genotype-related differences in pallidal oscillatory activity.^36^ To control for correct lead placement in the GPi, DBS electrodes were localised using Lead-DBS v3.0 for all patients as previously described.^10^^,^^37^ Preoperative and postoperative imaging were co-registered and non-linearly warped to MNI space (ICBM 2009b Nonlinear Asymmetric) using Advanced Normalisation Tools (ANTs, https://stnava.github.io/ANTs/). Following brain shift correction due to possible pneumocephalus, electrode trajectories were reconstructed using PaCER^38^ in case of postoperative CT images or TRAC/CORE^39^ if postoperative MRI scans were available.

**Table 1:** Clinical characteristics.

Patient	Type	Syndrome	Side	Dis dur	DBS dur	AIMS-item 7	Pre DBS	DBS OFF	DBS bipo	DBS mono	% bipo change	% mono change
1	CD	torti-, latero-, antecollis	right	24	186	0	25	17	13	4	23.53	84
2	CD	torticollis, head tremor	left	24	154	0	14	8	9	11	−12.50	21.43
3	SD	torticollis, left upper extremity	left	18	181	1	24	14	4	7	71.43	70.83
4	CD	torticollis, head tremor	right	10	9	2	14	12	8	10	33.33	28.57
5	CD	torti-, latero-, antecollis	right	23	167	0	18	10	5	7	50.00	61.11
6	CD	torticollis, laterocollis	right	4	14	0	19	17	18	15	−5.88	21.05
7	SD	torticollis, right shoulder girdle	right	21	91	3	15	18	13	12	27.78	20
8	SD	torticollis, left upper extremity	left	16	154	2	9	8	9	9	−12.50	0
9	CD	torticollis, laterocollis	left	25	195	0	22	13	7	15	46.15	31.82
10	SD	torticollis, left upper extremity	left	30	133	0	20	19	11	10	42.11	50
11	CD	torticollis, antecollis	right	21	9	4	25	21	20	16	4.76	36
12	CD	torticollis, oro-mandibular	left	9	41	1	20	25	3	4	88.00	80
13	CD	torticollis, laterocollis	left	14	11	1	17	14	11	13	21.43	23.53
14	CD	torticollis, head tremor	left	18	13	3	16	13	9	10	30.77	37.5
15	CD	torticollis	left	6	16	2	7	12	11	6	8.33	14.29
**Mean**			**9 left**	**18**	**91**	**1.25**	**18**	**15**	**10**	**10**	**27.78**	**37.51**
**Std**			**5 right**	**8**	**75**	**1.29**	**5**	**5**	**5**	**4**	**29.22**	**26.16**

CD = cervical dystonia, SD = segmental dystonia, dis dur = disease duration, DBS dur = DBS duration.

All patients included in the study had a chronic DBS setting with a monopolar montage (see Table 1 and Supplementary Table S1). Prior to MEG-recordings, an experienced clinician (BHB, RL, LG) established a bipolar stimulation setting with an amplitude about 30%^40^^,^^41^ higher than the monopolar setting aiming to elicit a comparable clinical effect based on the individual target symptom for each patient. This step was necessary to reduce DBS-artifacts in the MEG recordings, since the artifacts induced by bipolar DBS are around 25 times lower in amplitude than those induced by monopolar DBS.^40^, ^41^, ^42^ For the bipolar montage, the anode was moved to the contact dorsal to the active cathode. Exceptions were made when the active cathode was the uppermost contact (as in subjects 3, 8 and 11), or when the setting was not tolerated (Subject 1, 4, 6, 9 and 12), in which case alternative bipolar configurations were tested (see Supplementary Table S1). After at least 30 min of stimulation with the newly established bipolar setting, the motor part of the Toronto Western Spasmodic Torticollis Rating Scale (TWSTRS) was assessed by video. TWSTRS scores for chronic, monopolar stimulation settings and preoperative scores had to be retrospectively scored from archival videos, since time constraints did not allow acquisition on the same day of recordings. To account for mobile symptoms not captured by the TWSTRS, we re-evaluated all stimulation-off videos using item 7 of the Abnormal Involuntary Movement Scale (AIMS).^43^ Additionally, we examined bradykinesia specific subitems of the Unified Parkinson's disease Rating Scale, part III (UPDRS-III) assessing the amount of potential stimulation-induced slowness of movement (sub-items: 3.4–3.5 and 3.7–3.8).^23^ The first MEG recording was acquired with the bipolar stimulation setting activated. After the first recording the stimulation was switched off. During this time, patients were continuously assessed for a loss of stimulation effect and the stimulation off recording was started after (partial) reoccurrence of cervical dystonia or subjective discomfort (mean waiting time: 42 min; range: 30–120 min). After the MEG scan, the motor TWSTRS and UPDRS-III sub-items were videotaped again and rated offline from the video tapes, as stated above. All motor assessments were scored offline by movement disorder clinicians (LG, AM) blinded to the stimulation condition (“DBS bipo” and “DBS OFF” in Table 1). A paired t-test was used to evaluate changes in TWSTRS scores between the OFF-stimulation condition and bipolar DBS. The assumption of normality was verified using the Shapiro–Wilk test (>0.05).

### Ethics

Prior to study inclusion, informed written consent was obtained. The experimental protocols were approved by the local ethics committees of Charité—Universitätsmedizin Berlin (EA2/035/20) and the medical faculty at Heinrich Heine University Düsseldorf (no. 2021-1781). The study was conducted in accordance with the Declaration of Helsinki.^44^ Given extensive movement artifacts in the MEG recording, one patient had to be excluded (see below).

### Data acquisition

MEG resting state recordings were acquired for 5 min in both stimulation conditions (on and off DBS). Patients were instructed to keep their eyes open throughout the recording. MEG recordings were performed using a 125-channel-MEG system (Yokogawa ET 160, Tokyo, Japan) at the Physikalisch-Technische Bundesanstalt in Berlin and a 306 channel-MEG system (Neuromag, MEGIN Oy, Espoo, Finland) at the Institute of Clinical Neuroscience and Medical Psychology in Düsseldorf. In both MEG systems, data were recorded in the supine position. We purposefully chose to acquire data at the two centres in the supine position where in most patients with cervical dystonia there is considerable symptom relief.^45^ The MEG data were acquired with a sampling rate of 5 kHz, a hardware high-pass of 0.1 and a low-pass of 1 kHz (1.6 kHz in the Neuromag system). Heart beats and eye blinks were monitored using electrocardiography (ECG) and electrooculography (EOG) throughout recordings using an EEG amplifier synchronised with the respective MEG system.

### Data analysis

Analysing MEG recordings under active DBS is feasible, but requires careful consideration of DBS-induced electrical, cable-movement- and device-related artifacts.^32^^,^^33^^,^^42^ We therefore applied a combination of (1) a signal space separation method (tSSS)^46^ commonly used for MEG artifact reduction, which has been shown to reduce movement as well as electrical artifacts,^32^ (2) a spatial filter (linearly minimum constrained variance beamformer, LCMV) for source localisation^47^ additionally reducing movement related artifacts,^48^ and (3) a spectral method to reduce narrow band electrical artifacts at subharmonics and aliasing frequencies (Hampel-filter),^32^ that we applied on the source level.^32^ We provide exemplary data from two representative patients with movement-related artifacts before and after data cleaning was performed and data was reconstructed on the source level (Supplementary Fig. S1).

MEG data were analysed using Brainstorm.^49^ Data were visually inspected for channel jumps and the respective channels were marked as bad. One patient had to be excluded due to excessive movement artifacts that resulted in amplifier saturation in more than 50% of the channels (on average 6% of channels were rejected across all other recordings). This patient was the only one that had a Medtronic Activa PC with old Medtronic extension cables implanted underneath the clavicle. Half of the patients in the cohort had pulse generators implanted in the abdominal region (8/16) and the other half had a newer generation pulse generator by Medtronic or Boston Scientific (Percept PC or Vercise PC or Genus) implanted underneath the clavicle. The older Medtronic generator together with the old extension cables that contained more ferromagnetic components^40^ close to the sensors (left clavicle) could have resulted in stronger artifacts than in the rest of the cohort.^33^

Next, tSSS was applied to the data. After tSSS we detected remaining heartbeat and eye blink artifacts using the ECG and EOG signals and applied signal-space projection (SSP) as implemented in Brainstorm to reduce these artifacts where applicable. Data were visually inspected for remaining artifacts and the respective segments of data were rejected. Next, the data were down-sampled to 250 Hz and a high-pass filter at 3 Hz was applied. Individual preoperative MRIs were used to extract cortical surfaces using CAT 12.^50^ The surfaces were co-registered with the MEG recordings in Brainstorm using an iterative closest-point rigid-body registration. The forward model was generated using an overlapping spheres head model. Then, the data were localised to these individual cortex source grids (15,000 vertices) using LCVM beamforming.^47^ The cerebellum was not completely depicted in most of the individual MRIs. Imaging was acquired preoperatively, and some patients had been operated several years before (see results), so no standardised imaging protocol was applied across all patients and the two centres. Given the missing depiction of the cerebellum, we repeated the analysis, reconstructing the cerebral and cerebellar source time series directly onto the *surface*-based MNI template space (ICBM 2009c Nonlinear Asymmetric) instead of the individual anatomy. We repeated the source localisation and subsequent analysis using a *volumetric* source model to capture deeper cerebellar sources. After source localisation we applied the Hampel filter to reduce narrow-band electrical artifacts at the subharmonics and aliasing frequencies of the stimulation frequency.^33^

**Fig. 1:**
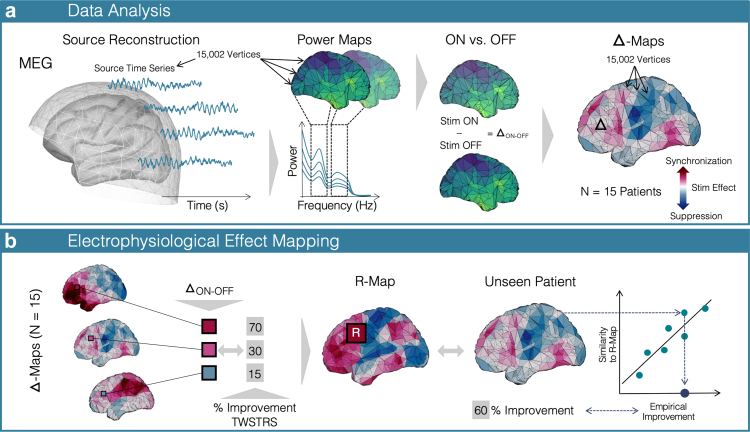
**Methods.** (A) Cortical source time series were reconstructed and localised on to a template brain. Power spectra were computed for each individual vertex resulting in a power map for each of the two frequency bands studied in each stimulation condition. The power maps for the DBS OFF recording were subtracted from the DBS ON recording to generate Δ-maps for each patient in the low-frequency and beta band. In the Δ-map, positive values represent a synchronisation (red), and negative areas represent a DBS-induced suppression. (B) In each vertex of the Δ-maps, we correlated the power change with the percentage improvement in the TWSTRS motor scale across all patients. This results in a correlation map (R-map) in which each vertex represents the correlation coefficient of DBS improvements and changes in power in this particular vertex across the cohort. This map represents an optimal pattern of change associated with a beneficial stimulation setting. Thus, the spatial similarity of an individual map with the R-map is an estimate of the DBS effect. To validate the R-map, it is generated leaving out one patient and spatially correlating the R-map with this individual pattern to estimate this patient's improvement. The correlation of the resulting spatial similarity with the actual (empirical) clinical improvements is then assessed to test how robust the model is.

After filtering the data, we z-scored the cortical source time series^49^ and computed the power spectrum densities using Welch's method (window length: 4 s, 50% overlap). We then applied a smoothing kernel with 6 mm and normalised the spectrum by dividing it by the power at higher frequencies (55–95 Hz). Next, the data were projected to the MNI-space to enable group level analysis. For the analysis of cerebellar power changes, source localisation had already been performed in MNI space in the first step, so that all of the patient source data were already in the same space. The subsequent methodological workflow is depicted in Fig. 1. Each of the cortical power spectra was averaged in two power bands of interest (1) the low frequency band (6–12 Hz) and (2) the beta band (13–30 Hz) resulting in a total of 15 power maps per stimulation condition and frequency band respectively (Fig. 1A). The lower border of the low-frequency band was deliberately chosen at 6 Hz to ensure an adequate distance to the previously applied high-pass filter (Supplementary Fig. S2A). Next, the stimulation OFF power maps were subtracted from the stimulation ON maps to generate two sets of 15 maps depicting the difference between stimulation conditions (Δ-map) in the chosen frequency bands, respectively. Higher values in the Δ-map signify an increase (power ON > OFF) and lower values a suppression of power (ON < OFF) in the respective frequency band induced by DBS (Fig. 1A).

### Statistics

First, to assess the magnitude of DBS-induced power changes on the group level and their relationship to clinical outcomes in certain *regions of interest* (ROI), we performed a paired t-test across the 15 cortical power maps ON vs. OFF stimulation. Based on the un-corrected significant vertices (P < 0.05) we selected one region of interest in each of the two t-maps with the highest t-values and the largest number of connected vertices (low-frequency and beta). Regions of interest were defined as a region including all these connected vertices. We then extracted the mean power differences in these regions of interest and correlated each of them with the % change in TWSTRS (percentage improvement (OFF-ON)/OFF, since larger scores are expected in OFF-stimulation condition) and bradykinesia subitems of the UPDRS-III respectively (percentage change (ON-OFF)/ON, since larger scores might occur in the ON- rather than the OFF-stimulation condition). Two-tailed correlation tests (Spearman's rho) were applied and P-values were corrected for multiple comparisons across regions of interest and frequency bands using Bonferroni correction. In a supplementary analysis, the cohort was split into responders and non-responders based on the clinical testing with bipolar DBS (see above) and the paired t-test across the cortical low-frequency power maps ON vs. OFF stimulation was applied for each of the two sub-cohorts separately to compare the cortical patterns of change in the low-frequency band.

Second, to map the individual DBS outcome to oscillatory power changes across the *whole cortex*, we correlated the low-frequency power changes in each vertex of the Δ-map with DBS outcomes (% improvement in TWSTRS score, see above) across the cohort (Fig. 1B). The resulting map of correlation coefficients (R-map) represents the pattern of changes across the *whole cortex* that was associated with an optimal DBS outcome.^34^ Next, the R-map was generated again, each time leaving out one patient and spatially correlating this particular patient's Δ-map with the respective R-map. Assuming that the R-map represents the optimal cortical pattern of oscillatory power changes, the spatial similarity of R-map and individual Δ-map can serve as an estimate of DBS outcome in the respective patient. To test how accurate these estimates were, we correlated them with the empirical DBS outcomes (Fig. 1B). We repeated this analysis using two- and four-fold cross-validations, randomly dividing the data into two or four equal folds to estimate improvements based on the R-map generated in the rest of the data. Given that for these cross-validations, only positive correlations are meaningful, one-sided (right-tailed) significance tests were applied for the correlation (Spearman's rho).^51^

Finally, in an exploratory analysis on the contribution of cerebellar power changes to DBS outcome across the cohort, we applied the same mapping approach (see Fig. 1B) to the source level power maps including the cerebral *and* cerebellar cortex. As mentioned above, these maps could not be generated using the subject-specific anatomy, which is why we focused on the cerebral cortex as a primary analysis.

The sample size was determined by practical constraints, given the small number of patients with cervical dystonia undergoing DBS. Some of our previous MEG or EEG studies had sample sizes around 10.^5^^,^^22^ Post-hoc sensitivity analyses showed that with our final sample of N = 15, the study had 84% power to detect a one-sided Spearman's rho of 0.73. The minimal detectable effect size (80% power, α = 0.05) was estimated as rho = 0.616. Both are considered large effect sizes.

### Role of funders

The funders had no role in study design, data collection, data analyses, interpretation, or writing of report.

## Results

### Low frequency power suppression correlates with DBS outcome

**Fig. 2:**
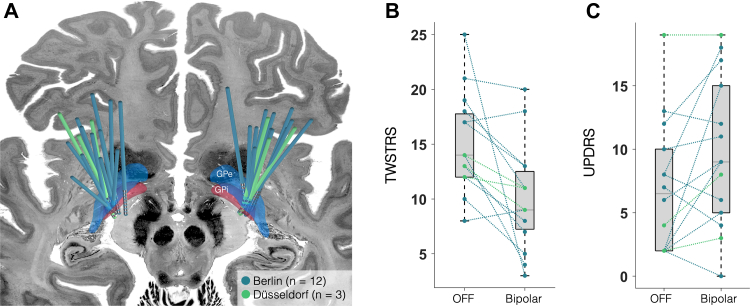
**Stimulation effects and electrode placement.** (A) Electrode localisation for both, the patient cohort from Berlin (n = 12, blue) and the cohort from Düsseldorf (n = 3, green). Electrodes were placed in the posterolateral GPi. (B) Comparison of mean TWSTRS scores between the bipolar, and off stimulation conditions. (C) Comparison of bradykinesia items of the UPDRS without (OFF) and with bipolar DBS.

For MEG recordings we switched all 16 patients from the established chronic monopolar setting to a bipolar stimulation setting to reduce DBS-induced artifacts.^40^, ^41^, ^42^ After at least 30 min of bipolar stimulation, the motor part of the Toronto Western Spasmodic Torticollis Rating Scale (TWSTRS) was assessed by video. There was a mean TWSTRS change of 5 points between the off-stimulation condition and the bipolar GPi stimulation on the day of MEG recordings (TWSTRS OFF vs. bipolar ON: 15 ± 5 vs. 10 ± 5 points) and a significant overall clinical response to the bipolar GPi stimulation compared to the off-stimulation condition (t (14) = 3.12; P = 0.008; Cohen's |d| = 0.81). On an individual basis, ten patients showed a change of at least 3 points, which is considered a minimal clinically important difference based on the definition by Espay et al.,^52^ while five patients showed a smaller difference (less than 20% between ON and OFF condition) and were considered as non-responders. Electrode localisation confirmed the accurate placement of DBS electrodes in the posterolateral GPi (Fig. 2).

**Fig. 3:**
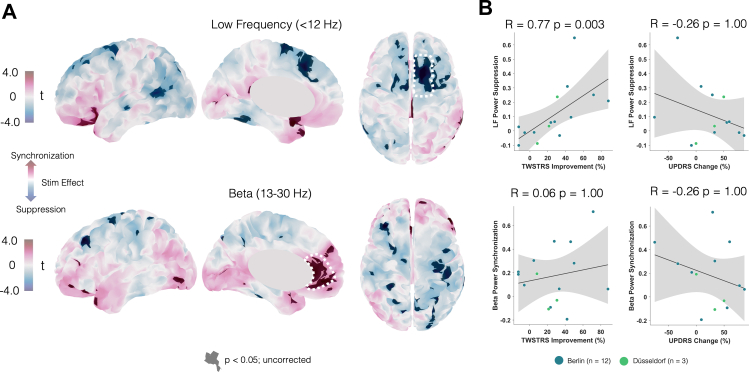
**Stimulation induced cortical changes relate to DBS outcome.** (A) T-maps of the power changes in low-frequency and beta band with red colours indicating a synchronisation and blue colours indicating a suppression in power induced by DBS. Darker colours mark vertices with uncorrected P-values <0.05 that served for visualisation and to identify regions of interest. The circled areas were identified as regions of interest based on the largest number of connected vertices with the highest t-values. (B) Mean low frequency power suppression in the SMA and beta power synchronisation in the cingulate cortex were correlated with TWSTRS improvements and the change of UPDRS bradykinesia items respectively. Grey shaded areas represent the 95% confidence interval. P-values were Bonferroni-corrected for multiple comparisons across regions of interest and scores.

To assess the magnitude of DBS-induced power changes on the group level and their relationship to clinical outcomes in certain *regions of interest* (ROI), we performed a paired t-test across the 15 cortical power maps ON vs. OFF stimulation. DBS induced cortical power was suppressed in motor areas, with low-frequency power suppression in the SMA and motor cortex, and beta power suppression mainly in sensorimotor cortices (Fig. 3). There were two main regions that stood out in the thresholded maps with low-frequency suppression in the right SMA and beta synchronisation in the cingulate cortex. When correlating the right SMA low-frequency suppression and cingulate cortex beta synchronisation with the percentage change in TWSTRS and UPDRS bradykinesia items, we found a significant correlation between TWSTRS improvement and low-frequency suppression in the right SMA (R = 0.77, P = 0.003). Low-frequency suppression did not significantly correlate with UPDRS changes (R = 0.26, P = 1.00). Beta synchronisation was neither associated with TWSTRS nor UPDRS changes (TWSTRS: R = 0.06, P = 1.00; UPDRS: R = −0.26, P = 1.00).

Previous work has defined low-frequency oscillations within varying bands, spanning 4–10 Hz in one of the seminal studies by Silberstein et al.,^19^ subsequently 4–12 Hz, e.g. by Barow et al.^5^ and Neumann et al.^18^ We therefore repeated the primary analyses using a frequency band definition of 4–12 Hz and results remained similar (R = 0.75, P = 0.001; Supplementary Fig. S2B).

To further investigate the differences in low-frequency power changes in patients that responded or did not respond well to stimulation, we computed the low-frequency t-maps for responders and non-responders (<20% improvement, see above) separately. While responders displayed a similar low-frequency power suppression in SMA and motor cortex, the non-responder t-map was dominated by low-frequency power synchronisation in parietal and motor areas (Supplementary Fig. S3).

### Whole-cortex signature explains variance in DBS outcomes

In dystonia patients treated with pallidal DBS, outcomes are often variable, and this is also the case in our patient cohort, ranging from 12% deterioration to 88% improvement during bipolar stimulation. Together with the relatively small sample size, this might have prevented us from observing a robust acute group effect for cortical power changes (Fig. 3). Therefore, we weighted the power changes by the individual DBS effect, applying an established mass-univariate approach^34^^,^^35^^,^^53^ (Fig. 1B). As described in the method section, we built an R-map by vertex-wise correlation of low-frequency power changes and individual TWSTRS improvements due to DBS (Fig. 1B). This highlights changes, which would have been most beneficial across patients within our cohort. We focus on the DBS improvement in motor symptoms and respective changes in low-frequency activity since no correlation with beta activity was shown in the ROI analysis.

**Fig. 4:**
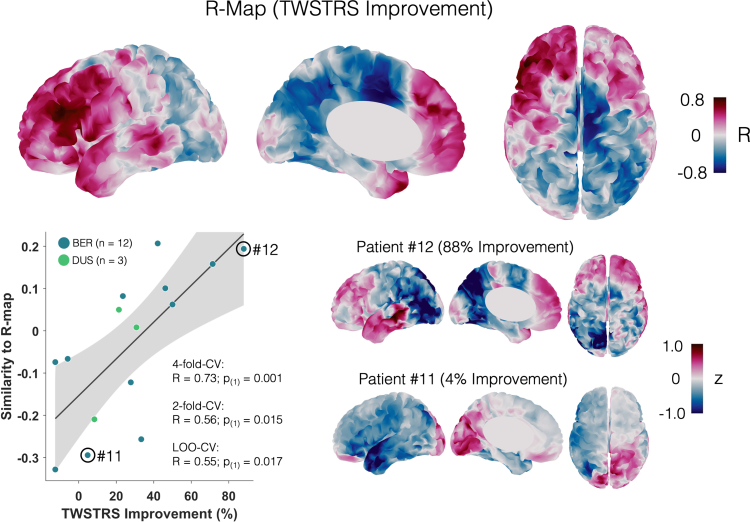
**Optimal electrophysiological response map (R-map).** Low-frequency changes and TWSTRS improvement negatively correlate in motor areas and positively in prefrontal areas. Spatial similarity of individual patient maps to the R-map correlates with the percentage improvement in TWSTRS motor scores (correlation plot depicts 4-fold-cross-validation results). The grey shaded area represents the 95% confidence interval. Δ-maps of a top responder (#12) and poor responder (#11) are shown next to the correlation plot.

The R-map showed a pattern of low-frequency suppression in the SMA and mesial motor cortex as well as frontal synchronisation, generally aligning with the overall pattern of change observed in the t-map. Indeed, this pattern is reflected well in the case of a top-responder (patient # 12, see Fig. 4) and appears almost inverted for a non-responder (patient # 11, see Fig. 4). Interestingly, when comparing the electrode placements of patient 11 and 12, the responder electrodes were both inside the GPi, while the non-responder electrodes were slightly medial/lateral to the GPi (Supplementary Fig. S4). When correlating each patient's difference map with the R-map using different cross-validation designs, the model explained significant amounts of variance in DBS outcomes (four-fold cross-validation: R = 0.73, P_(1)_ = 0.001; two-fold cross-validation: R = 0.56, P_(1)_ = 0.015; leave-one-out design: R = 0.55, P_(1)_ = 0.017).

### DBS-induced cerebellar and cerebral changes

**Fig. 5:**
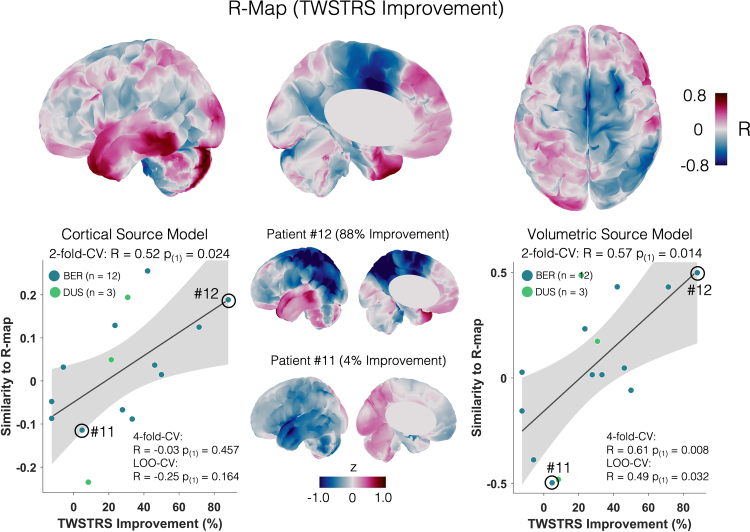
**Optimal electrophysiological response maps including the cerebellum.** Correlation map (R-map) with negative correlations of low-frequency changes in motor areas and positive correlations in prefrontal areas and the cerebellum. Similarities to the R-map correlate with the percentage improvement in TWSTRS motor scores for a cortical and a volumetric source model (correlation plots depict results from 2-fold-cross-validations). The grey-shaded areas represent the 95% confidence intervals. Δ-maps of a top responder (#12) and poor responder (#11) are shown next to the correlation plots.

Based on previous findings, we also expected changes in low-frequency power in the cerebellum.^10^^,^^15^^,^^16^^,^^22^ Importantly, this analysis was performed with a template brain, due to missing depictions of the whole cerebellum in some of the preoperative imaging in our cohort (see methods). Again, the resulting R-map had a similar pattern of power changes with suppression in SMA and motor cortex, while cerebellar sources mainly showed a synchronisation of low-frequency power (Fig. 5). Subjecting the R-map to cross-validations yielded significant results for the two-fold cross-validation (R = 0.52, P_(1)_ = 0.024). We repeated the analysis using a volumetric source model to potentially capture deeper cerebellar sources (see Supplementary Fig. S5) and cross-validations again yielded significant results (four-fold-cross-validation: R = 0.61, P_(1)_ = 0.008; two-fold-cross-validation: R = 0.57, P_(1)_ = 0.014; leave-one-out-cross-validation: R = 0.49, P_(1)_ = 0.032).

To assess the influence of other covariates, typically relevant for DBS outcome in patients with dystonia, like age, disease duration and disease severity (Isaias et al., 2011), we built a linear model with TWSTRS improvement as dependent variable and similarities to the R-map (4-fold-CV), age, disease severity and disease duration as independent variables to check for the variance explained by each of these patient characteristics. In this model, similarity to the R-map was the only significant predictor of DBS outcomes and there were no significant interaction terms (similarity to R-map (4-fold-CV): β_std_ = 0.69, P = 0.014; age: β_std_ = 0.17, P = 0.451; disease duration: β_std_ = −0.19, P = 0.439; disease severity: β_std_ = 0.19, P = 0.405). To rule out potential effects of disease type (cervical vs. segmental dystonia), a one-way ANOVA was performed with TWSTRS improvement was dependent and disease type as independent variable. There was no main effect of disease type on TWSTRS improvement (F (1, 13) = 0.12, P = 0.738). To account for mobile symptoms not captured by the TWSTRS, we fitted another linear model, with TWSTRS improvement as the dependent variable and both similarity to the R-map and AIMS item 7 scores as predictors. Including AIMS item 7 in the model revealed no significant effect or interaction related to mobile symptoms, while the similarity to the R-map remained a significant predictor (4-fold CV: β_std_ = 0.30, P = 0.001; AIMS: β_std_ = 0.13, P = 0.103).

## Discussion

Here, we investigate the effects of pallidal DBS on oscillatory network activity in 16 patients with cervical dystonia. We showed that, first, pallidal neurostimulation induces changes in cortical oscillatory activity with low-frequency suppression in the SMA being associated with clinical improvements. Second, the overall pattern of DBS-induced low-frequency power changes explained significant amounts of variance in clinical DBS effects across patients. Third, low-frequency synchronisation in the postero-mesial cerebellar cortex along with the overall cerebro-cerebellar pattern of changes appears to be relevant for DBS outcome as well. Our study sheds light on the whole-cortex effects of pallidal DBS emphasising low-frequency suppression in SMA and motor cortex, as well as low-frequency synchronisation in frontal and cerebellar regions as key network mechanisms of effective neurostimulation in patients with cervical dystonia.

Cortical and subcortical low-frequency activity has been described as a pathophysiological hallmark in dystonia.^3^^,^^5^^,^^17^, ^18^, ^19^^,^^22^ Low-frequency oscillations, however, constitute a broader phenomenon within the basal ganglia–thalamo–cortical loop and are observed across different hyperkinetic disorders, such as dystonia, tic disorders, or levodopa-induced dyskinesia.^3^^,^^18^^,^^54^, ^55^, ^56^, ^57^, ^58^, ^59^, ^60^ Similarly, low-frequency power in LFP recordings from GPi, striatum, STN and thalamus has been linked to hyperkinetic symptom severity.^3^^,^^57^^,^^59^ Recent work suggests that the exaggerated LF activity associated with symptom severity in dystonia may originate within the basal ganglia network itself—specifically in striato-pallidal coupling. Such enhanced LF coupling could reflect an imbalance in the direct pathway, a mechanism proposed as a pathophysiological correlate of dystonia.^3^ The suppression of this activity, in turn, has been suggested as a potential mechanism of DBS in these patients.^5^^,^^6^ Indeed, our results support this notion through the correlation between low-frequency power suppression in the SMA and DBS-induced clinical improvement in our cohort (Fig. 3).

Anatomically, our study highlights the role of the premotor cortex in dystonia and the effectiveness of DBS. The disruption in sensory-motor integration within the premotor cortex and particularly the SMA may play a critical part in the pathophysiology of dystonia.^61^ Excessive activation in sensorimotor cortex and SMA is present in patients with dystonia,^62^ and is suppressed by GPi stimulation.^25^, ^26^, ^27^, ^28^, ^29^, ^30^ Therefore, one proposed mechanism of action is that DBS indirectly increases thalamocortical inhibition.^63^ In our study, LF modulation at the cortical level appears broadly distributed across the frontal cortex. Therefore, the cortical changes identified by our model are most likely driven by upstream influences via the direct pathway and mediated through the thalamus, which projects widely to these frontal regions.

In our study, the R-map has negative peaks in SMA and motor cortex. The stronger the low-frequency suppression, the better the clinical DBS effect. At the same time, we see positive peaks in frontal areas like the inferior frontal gyrus, the prefrontal cortex as well as the cerebellum, that reflect an increase in low-frequency activity positively associated with the DBS effect. Thus, while low-frequency suppression occurs in cortical motor areas, low-frequency synchronisation is present within the prefrontal network and the cerebellum, and together this pattern represents the optimal response signature for pallidal DBS in cervical dystonia. In previous work, cerebellar connectivity was negatively associated with motor activation recorded in the SMA in manifesting vs. non-manifesting dystonia.^64^ This fits well with our data showing cerebellar low-frequency synchronisation in parallel with suppression of low-frequency activity in the SMA. Further, non-invasive cerebellar stimulation has led to short-term improvement in dystonia symptoms pointing towards a potential compensatory or protective effect of cerebellar activity.^65^ In accordance with this hypothesis, we previously showed that cerebello-pallidal alpha band coherence was inversely correlated with dystonic symptoms.^22^

Previous studies that applied a network mapping approach using normative fMRI connectivity between the stimulation site and cortex found that SMA connectivity positively correlated with the DBS outcome, while occipital and parietal connectivity were negatively correlated with outcomes.^10^^,^^11^ Thus, the functionally connected cortical areas relevant to DBS outcome partly overlap with the areas that show low-frequency suppression in our study.

We observed a slight lateralisation of the R-map to the right hemisphere, especially regarding low-frequency suppression in the SMA. Our cohort consisted of patients with cervical dystonia, and in a small subgroup of four patients, additional dystonic symptoms were observed in an adjacent limb (see Table 1). Three patients had appendicular dystonic symptoms in the left upper extremity. This could explain the slight lateralisation of the R-map to the contralateral (i.e. right) SMA and motor cortex. Additionally, the majority of patients with isolated cervical dystonia had left-lateralised symptoms (six patients vs. four patients with right-lateralised symptoms). In fact, symptom lateralisation in cervical dystonia is reflected by interhemispheric differences of low-frequency power in the GPi.^66^^,^^67^

In our thresholded t-map, beta power changes peaked in the cingulate cortex, but did not correlate with DBS-induced changes in UPDRS bradykinesia items or TWSTRS motor scores. Previous work has highlighted *local* pallidal beta power as a relevant marker for stimulation-induced slowness of movements.^23^ In our whole-*cortex* ROI analysis, we did not find this relationship. In previous work using cortical recordings of patients with Parkinson's disease, neither cortical beta power nor beta synchrony robustly correlated with symptom improvement.^68^^,^^69^ DBS-induced glucose uptake in the cingulate cortex has been attributed to cognitive DBS side effects in patients with dystonia, measured using a trail-making test.^31^ We did not test for any cognitive side effects of stimulation in our cohort and cannot rule out the possibility that the change in beta power in the cingulate cortex might be relevant for cognition in some of the participants.

DBS effects in patients with dystonia can be variable, and patient populations are small.^2^ Thus, designing a study to investigate the electrophysiological underpinnings of these variable effects on the group level is challenging by nature. We applied an established method to map these variable outcomes to the cortical patterns of DBS-induced change^11^^,^^34^^,^^35^^,^^53^ (Fig. 1). This method (1) leverages the cortical pattern rather than the absolute amount of change and (2) weights the changes by individual outcomes correlating the cortical pattern of changes with DBS outcomes across the cohort.^34^ Thus, this approach emphasises the pattern of change of top-responders and, therefore, reflects the signature that is relevant for an optimal DBS effect. Indeed, when dividing the cohort into responders (>20% TWSTRS improvement) and non-responders, we still see a clear motor cortex and SMA low-frequency suppression in the responder group, while non-responders appear to show low-frequency synchronisation in motor and parietal areas (Supplementary Fig. S3).

Future work could test the causal relevance of low-frequency oscillations within basal ganglia–cortical networks by applying lower frequency GPi stimulation to experimentally induce synchronised low-frequency activity. Such paradigms may help determine whether artificially enhancing low-frequency synchrony worsens dystonic symptoms or reproduces the network patterns observed in the DBS OFF state. Recent evidence that gamma activity at half the DBS frequency is entrained by DBS and associated with improved clinical outcome^70^ further supports the feasibility of frequency-specific network modulation to test causal hypotheses.

Our study has several clinical and research implications. The relationship we observe between SMA low-frequency suppression and DBS outcome could represent an easily accessible non-invasive (e.g. EEG or dryEEG, such as in this preprint^71^) marker for DBS programming or intraoperative targeting in dystonia. Additionally, the low-frequency suppression in the SMA might represent an accessible target signal for closed-loop DBS or non-invasive stimulation paradigms. Lastly, the optimal response network we identified could inform multifocal tDCS montages for non-invasive network stimulation.^51^

Several limitations have to be considered in the context of our study. First, our DBS ON vs. OFF recordings were both on the same day, and therefore, we can only assess the relatively acute clinical effects of the stimulation. Thus, the effect we measure could be smaller given a prolonged DBS effect after deactivation of the stimulation. Additionally, the same sequence of stimulation conditions (bipolar DBS, then stimulation off) was used across participants. Thus, some effects might not have completely resolved after deactivating the stimulation and some effects might not yet have built up before the experiment started. Carry-over effects are possible and might have influenced the clinical state in each of the two conditions. However, this was partly mitigated by the fact, that we contrasted the two conditions, assessing the acute electrophysiological as well as clinical *changes*. Second, we applied an experimental bipolar stimulation setting to reduce DBS-induced artifacts in the MEG that might not have reflected the chronic DBS effect in some patients. In fact, three patients that showed a small to moderate response to monopolar DBS did not respond to the experimental bipolar setting (patients 2, 6 and 11) and one patient showed a much smaller effect during bipolar stimulation (patient 1) (Table 1). However, previous work has shown, that the current electrophysiological read out of GPI low frequency power closely relates to the current clinical state.^17^ Thus, our experimental setup most likely reflects short term modulation of DBS-induced network effects in parallel with the respective changes in clinical state. Third, the method we applied to map DBS effects to electrophysiological change signatures requires future replication in other disorders and larger patient populations to validate our findings and methodology. Fourth, we performed the study using two different MEG devices and at two different DBS centres. To rule out centre-specific bias, we focused on harmonising the study protocols and recording conditions as much as possible. Before study initiation we performed a DBS phantom study in both MEG systems and artifact contamination and data quality across MEG devices was comparable.^33^ We further harmonised acquisition conditions, e.g. we used a supine recording setup in both MEG scanners. Additionally, since analyses were performed on the source level, there is a certain degree of standardisation across devices and subjects. Furthermore, absolute signal magnitudes were less relevant in the network mapping approach, because the pattern of change rather than the absolute magnitude of changes accounts for the spatial similarity used as estimate for individual improvements.^34^ Finally, there were inconsistent imaging protocols across the two centres, leading to incomplete depiction of the cerebellum in some patients and the need for an analysis in standard anatomical space. When comparing the results between the individual and standardised anatomical spaces, we observe smaller correlation coefficients in the cortical areas shared across the two analyses for the standard template. This implies, that we tend to generally underestimate the effects in the MNI template analysis and the cerebellum. In fact, for forward modelling using MEG the difference between normative vs. individualised anatomy is much smaller than for EEG, since layer- and head shape do not play a significant role for the sphere-based forward model in MEG source reconstruction.^72^^,^^73^

In conclusion, we identify a distinct cortical and cerebellar signature of low-frequency power changes to pallidal neurostimulation in patients with cervical dystonia that correlates with clinical DBS outcome. When weighting the cortical signatures using individual DBS outcomes, we observed low-frequency power reduction over motor areas and power increase in cerebellar and frontal areas that was associated with optimal symptom relief. These findings re-emphasise dystonia as a network disorder with low-frequency activity as a potential biomarker for dystonic motor symptoms. Moreover, within this network, DBS may lead to a suppression of pathologically enhanced low-frequency activity at the SMA as well as motor cortical level and at the same time induce activation of potential compensatory effects via enhancing cerebellar low-frequency activity. Electrophysiological signatures of DBS network effects might inform clinical programming and DBS targeting in the future.

## Contributors

BHB and RL contributed equally to conceptualisation, data curation, formal analysis, investigation, methodology, visualisation, and writing the original draft. HV, ALM, LLG, LF, and THS contributed to investigation, formal analysis, validation, methodology and writing–reviewing and editing. AS contributed to conceptualisation, funding acquisition, investigation, supervision, and writing–reviewing and editing. EF and AAK contributed equally to project administration, resources, data curation, conceptualisation, funding acquisition, investigation, supervision, and writing–reviewing and editing. All authors read and approved the final version of the manuscript. BHB, RL, HV, EF and AAK have accessed and verified the underlying data.

## Data sharing statement

The data that support the findings of this study are available upon reasonable request from the corresponding author (andrea.kuehn@charite.de). The data are not publicly available, given that they contain identifiable information that could compromise the privacy of patients.

## Declaration of interests

AAK has received consulting fees and honoraria for talks from Medtronic, Boston Scientific, Zambon, Stada Pharm and Ipsen. AS received consulting fees and honoraria for talks from Medtronic, Boston Scientific and Abbott. LKF received honoraria for talks from Medtronic. RL received honoraria for talks from University Zurich, International Movement Disorders Society, Swiss Movement Disorders Society and Deutsche Gesellschaft für Klinische Neurophysiologie.
