# Supplementary material for: Spatial signature of low-frequency network changes accounts for pallidal stimulation outcome in cervical dystonia
Source: eBioMedicine. 2026 Jan 28;124:106140. doi: 10.1016/j.ebiom.2026.106140 (PMC12905622; doi:10.1016/j.ebiom.2026.106140)
Supplement: Supplementary Figures and Table [file mmc1.docx]

**Supplementary Material**

|  | Bipolar Settings | | Monopolar Settings | |
| --- | --- | --- | --- | --- |
| Patient | L GPI | R GPI | L GPI | R GPI |
| 1 | 1+/2−, 4.2 mA, 60 µs, 180 Hz | 3+/2−, 4.2 mA, 60 µs, 180 Hz | C+/1−2−, 3.2 V, 60 µs, 180 Hz | C+/2−3−, 3.2 V, 60 µs, 180 Hz |
| 2 | 3+/2−, 3 V, 90 µs, 60 Hz | 3+/2−, 5.2 V, 90 µs, 60 Hz | C+/2−, 2.2 V, 90 µs, 60 Hz | C+/2−, 4 V, 90 µs, 60 Hz |
| 3 | 3+/4−, 3.6 V, 60 µs, 75 Hz | 3+/2−, 4.0 V, 60 µs, 75 Hz | C+/4−, 2.1 V, 60 µs, 75 Hz | C+/2−, 2.6 V, 60 µs, 75 Hz |
| 4 | 2+/3−, 4.5 mA, 90 µs, 130 Hz | 2+/3−, 1.0 mA, 90 µs, 130 Hz | C+/2−3−4−, 1.5 mA, 90 µs, 130 Hz | C+/2−3−4−, 1.5 mA, 90 µs, 130 Hz |
| 5 | 4+/3−, 4.2 V, 90 µs, 130 Hz | 4+/3−, 1.3 V, 90 µs, 130 Hz | C+/3−, 3.2 V, 90 µs, 130 Hz | C+/3−, 1 V, 90 µs, 130 Hz |
| 6 | 2+/3−, 2.2 mA, 90 µs, 130 Hz | 2+/3−, 2.5 mA, 90 µs, 130 Hz | C+/4−, 2.8 mA, 90 µs, 130 Hz | C+/4−, 2.8 mA, 90 µs, 130 Hz |
| 7 | 6+/5−, 4.6 mA, 120 µs, 231 Hz | 6+/5−, 4.6 mA, 120 µs, 231 Hz | C+/5−6−, 3.3 mA, 120 µs, 231 Hz | C+/5−6−, 3.3 mA, 120 µs, 231 Hz |
| 8 | 3+/2−, 3.6 V, 90 µs, 210 Hz | 3+/4−, 3.9 V, 90 µs, 210 Hz | C+/2−3−, 2.2 V, 90 µs, 210 Hz | C+/4−, 2.4 V, 90 µs, 210 Hz |
| 9 | 1+/2−, 5.9 V, 90 µs, 180 Hz | 2+/3−, 6.3 V, 90 µs, 180 Hz | C+/3−, 4.4 V, 90 µs, 180 Hz | C+/3−, 4.7 V, 90 µs, 180 Hz |
| 10 | 10+/9−, 4.2 V, 90 µs, 180 Hz | 2+/1−, 3.5 V, 90 µs, 180 Hz | C+/9−10−, 3.2 V, 90 µs, 180 Hz | C+/1−2−, 2.7 V, 90 µs, 180 Hz |
| 11 | 1+/2−, 2.4 mA, 90 µs, 159 Hz | 3+/4−, 1.8 mA, 90 µs, 159 Hz | C+/2−, 1.8 mA, 90 µs, 159 Hz | C+/4−, 1.4 mA, 90 µs, 159 Hz |
| 12 | 2+/3−, 4.6 mA, 90 µs, 185 Hz | 3+/2−, 5.2 mA, 90 µs, 185 Hz | C+/3−, 3.0 mA, 90 µs, 185 Hz | C+/3−, 3.0 mA, 90 µs, 185 Hz |
| 13 | 3+/1−2−, 4.5 mA, 90 µs, 125 Hz | 4+/2−3−, 4.5 mA, 90 µs, 125 Hz | C+/1−2−, 3.4 mA, 90 µs, 125 Hz | C+/2−3−, 3.3 mA, 90 µs, 125 Hz |
| 14 | 3+/1−2−, 3.1 mA, 60 µs, 210 Hz | 4+/2−3−, 3.1 mA, 90 µs, 210 Hz | C+/1−2−, 2.0 mA, 60 µs, 210 Hz | C+/2−3−, 2.0 mA, 90 µs, 210 Hz |
| 15 | 4+/3−, 5.4 mA, 90 µs, 100 Hz | 3+/2−, 5.4 mA, 90 µs, 100 Hz | C+/3−, 3.0 mA, 90 µs, 100 Hz | C+/2−, 3.0 mA, 90 µs, 100 Hz |

**Supplementary Table 1. Bipolar and monopolar DBS settings.** Stimulation settings for the left and right globus pallidus internus (L GPI, R GPI) in the bipolar and monopolar stimulation montages.

**Supplementary Figure 1. Artifact Reduction.** Two representative patient datasets with movement-related artifacts in the stimulation off condition from the two different MEG systems (P10: Yokogawa MEG; P14: Neuromag MEG). On the left raw MEG channel data is shown, on the right, data is shown after processing steps (tSSS, LCMV beamformer and Hampel filter). For visualization purposes, the source-level data on the right was extracted from the Desikan-Killiany atlas parcels, i.e. source time series were averaged across each atlas parcel using methods implemented in Brainstorm. In the raw data high amplitude, low-frequency movement-related artifacts can be observed in channels located on the side of the impulse generator, connector as well as extension cables. In patient 14 a slight tremor artifact can be seen in the channel level data, which is considerably reduced after data cleaning and projection to the source level.

**Supplementary Figure 2. Low frequency band definition. A** The primary analyses were focused on the frequency band between 6 and 12 Hz based on the grand average spectra that most consistently showed a peak in this range. Additionally, lower frequencies might have been influenced by the high-pass filter at 3 Hz. **B** We repeated the primary analysis using another frequency band definition with the lower frequency limit at 4 Hz. In the left panel the correlation between low-frequency power suppression in the SMA and TWSTRS improvement is shown, on the right the correlation between similarities to the R-map (4-fold-cross-validation) and TWSTRS improvement is depicted.

**
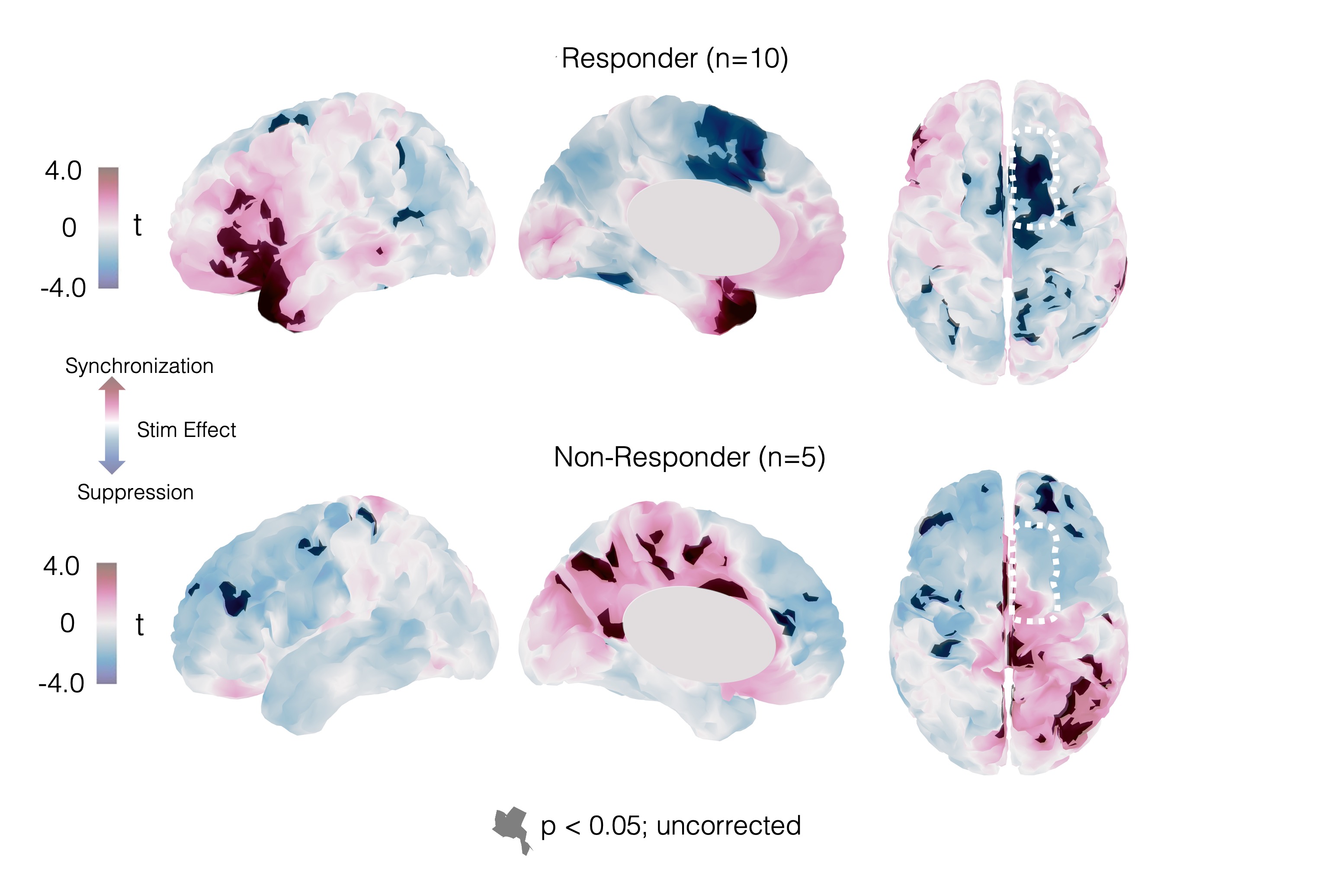
**

**Supplementary Figure 3. DBS-induced changes in Responders and Non-Responders.**T-maps showing the power changes in the low-frequency band with red colors indicating a synchronisation and blue colors indicating a suppression in power induced by DBS in responders (>20% TWSTRS improvement) and non-responders. The circled area was identified as region of interest in the primary analysis. These maps were again thresholded using the un-corrected significant vertices (*P*<0·05). In order to compare the pattern of low-frequency changes between responders (>20% TWSTRS improvement) and non-responders, we split the cohort into these two subgroups and ran a paired t-test across the respective cortical power maps ON vs. OFF stimulation in each of the two groups separately.

**
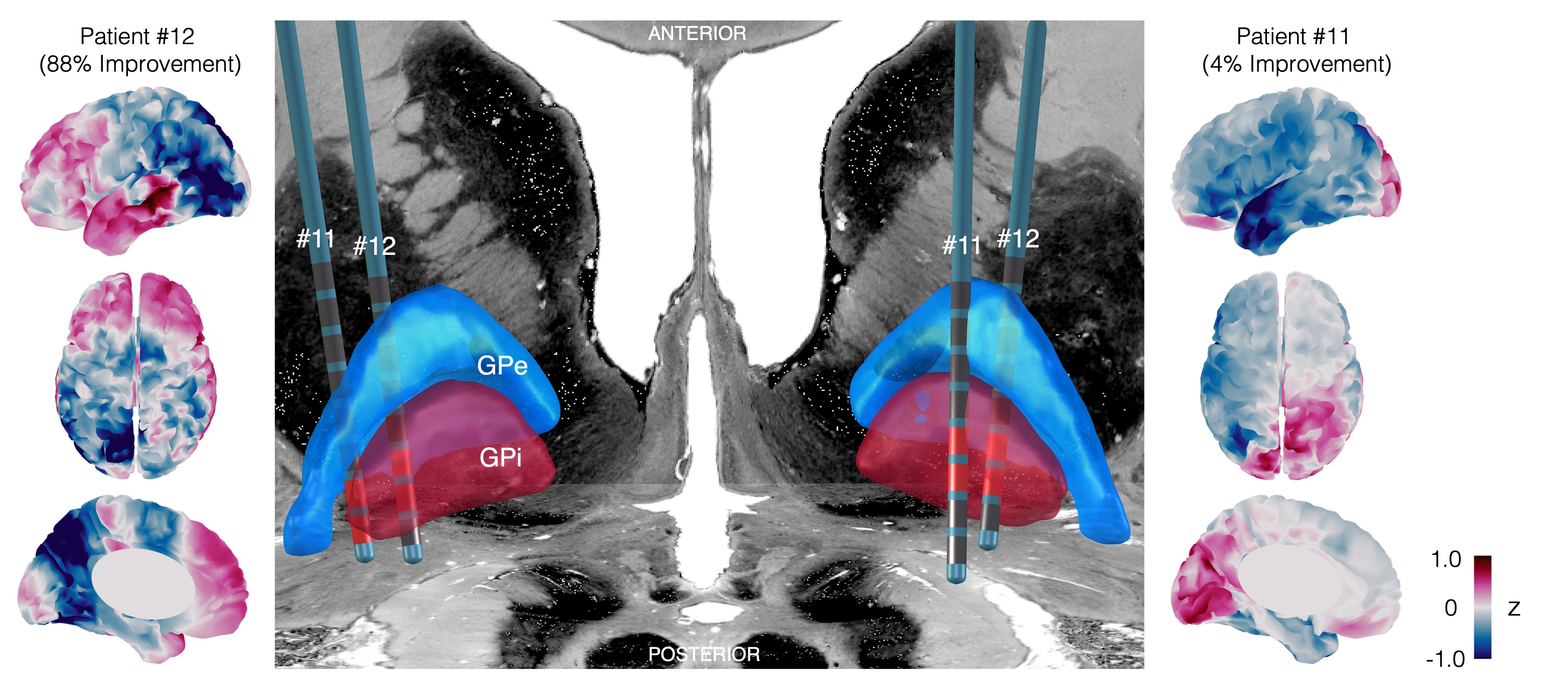
**

**Supplementary Figure 4. Electrode placement for top and poor responders.** Δ-maps of a top responder (patient #12) and poor responder (patient #11) are shown next to the anatomical scene, with DBS electrodes inside the GPi target region. The DBS electrode contacts active for bipolar DBS are highlighted in red. DBS electrodes for patient #11 (poor responder) are located slightly lateral (in left hemisphere) and medial (in right hemisphere) of the GPi, while the electrodes of patient #12 are both placed inside the GPi.


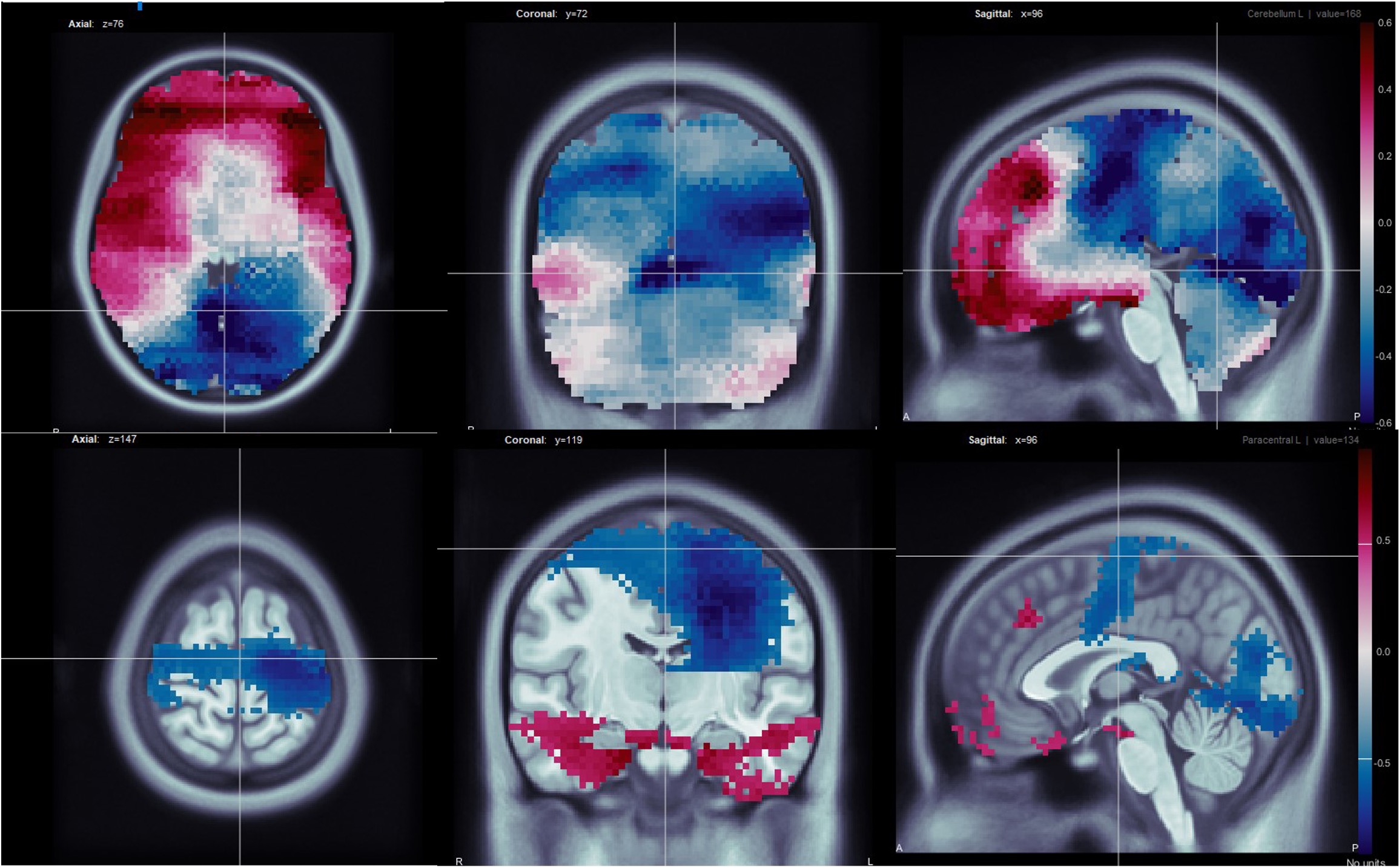


**Supplementary Figure 5.** R-map based on volumetric source models computed in template (MNI)-space.
